# Services That Matter for Aging in Place: Research on the Impact and Promise of Adult Day Centers

**DOI:** 10.1093/geroni/igaa057.2098

**Published:** 2020-12-16

**Authors:** Tina Sadarangani, Holly Dabelko-Schoeny

## Abstract

Adult day service centers (ADCs) in the United States are increasingly recognized as an important source of community-based long-term care for older adults. However, awareness, widespread utilization, reimbursements, and access to ADCs have been limited by a lack of evidence on ADCs’ impact. In this interdisciplinary symposium, we explore current research taking place in the realm of adult day services to understand the reach and impact of ADCs. We begin by examining the most current center-level and user-level data from the National Center for Health Statistics, and demonstrate how these data can be used to inform research and policy. We subsequently evaluate survey data from the National Adult Day Services Association that captures clinical data being collected in ADCs (N=250) surrounding users’ clinical outcomes. We then explore the effectiveness of four interventions on ADC users’ health and functional status: board games, cognitive behavioral therapy, aromatherapy and dance. Finally, we examine the association between adult day services use by African American persons with dementia and depressive symptoms in their caregivers. Our findings suggest that ADCs serve a complex population with high rates of poverty and chronic conditions, but ADCs can have a meaningful impact on users’ health and well-being by leveraging innovative programming. We conclude by discussing how standardization of data collection efforts could enable researchers and policymakers to evaluate ADCs’ impact and target funding towards services that maximizes users’ health and well-being.

